# Precision Medicine in Treating Lung Cancer: A Narrative Review on Treatments Targeting Oncogenic Genetic Mutations

**DOI:** 10.7759/cureus.108302

**Published:** 2026-05-05

**Authors:** Eesha Chitneni, Adwaith Venugopal, Confidence Obianuju Okorie, Annie T George, Aakriti Arya, Domenica Arias, Omar Yasin, Livia Cereser Prado de Souza, Roshan Afshan, Vyapti A Dave

**Affiliations:** 1 Medicine, MediCiti Institute of Medical Sciences, Hyderabad, IND; 2 Stroke Medicine, University Hospital of North Durham, Durham, GBR; 3 Internal Medicine, Northern Care Alliance NHS Foundation Trust, Royal Oldham Hospital, Oldham, GBR; 4 Medicine, Rajiv Gandhi University of Health Sciences, Kottayam, IND; 5 Medicine, Aarthi Eye Hospital, Karur, IND; 6 Medicine, National University of Loja, Loja, ECU; 7 General Surgery, Medway Maritime Hospital NHS Foundation Trust, Kent, GBR; 8 Medicine, Lusíada University Center, Faculty of Medical Sciences of Santos, Santos, BRA; 9 Internal Medicine, Detroit Medical Center, Detroit, USA; 10 Internal Medicine, Gujarat Medical Education and Research Society Medical College Valsad, Valsad, IND

**Keywords:** braf mutation, ca lung, egfr targeted therapy, kras mutation, non-small cell lung carcinoma (nsclc), small cell lung cancer (sclc), targeted anticancer therapy

## Abstract

Lung cancer is among the leading causes of cancer-related mortality worldwide. It is classified into small cell lung cancer (SCLC) and non-small cell lung cancer (NSCLC), with NSCLC being more prevalent. There have been advancements in managing extensive research on immunotherapy agents, oncolytic viruses, and targeted therapies by understanding the genetic mutations and molecular mechanisms behind lung cancer. Targeted therapy is the new paradigm in the treatment of lung cancer, especially NSCLC, and the aim is to inhibit specific proteins involved in cancer growth and progression. They primarily utilize small molecule inhibitors, monoclonal antibodies (mAbs), and antibody-drug conjugates (ADCs) to attack specific oncogenic driver mutations such as epidermal growth factor receptor (EGFR), anaplastic lymphoma kinase (ALK), Kirsten rat sarcoma viral oncogene homolog (KRAS), c-ros oncogene 1 (ROS1), and immune checkpoint pathways. These agents have improved outcomes and have shown efficacy in clinical trials, making them a preferred option over traditional chemotherapy. However, the associated adverse effects of these agents, along with drug resistance, remain a challenge. In this review, we discuss targeted therapies for lung cancer, their efficacy, clinical trials, adverse effects, mechanisms of resistance, and future directions.

## Introduction and background

Lung cancer remains the leading cause of cancer-related mortality globally, accounting for one in every five cancer-related deaths [1]. Due to its increasing prevalence, acquired resistance, today's ageing population, and late-stage diagnoses, lung cancer continues to pose a serious threat to global health [2]. The prognosis for lung cancer remains relatively poor despite advancements in screening, diagnosis, and therapy, especially for individuals with advanced illness. In 2022, it was found that in the United States, approximately $3,200 was received in funding from the National Cancer Institute (NCI) per patient death, which was significantly lower than that allocated to many other malignancies, including brain, prostate, and breast cancer [3].

Lung cancer is broadly classified into two major histological subtypes: small cell lung cancer (SCLC) and non-small cell lung cancer (NSCLC), with NSCLC accounting for approximately 85% of cases. They are categorized mainly based on their molecular and histological features [4]. Considering these different histological types of lung cancer, we understand that each histological type has different genetic profiling and sensitivity to treatment. Targeted therapy based on the patient's biological and genetic profile is a promising approach to optimize efficacy with the available agents [5].

Advances in molecular biology have demonstrated that many lung cancers are driven by specific genetic alterations that promote uncontrolled cell proliferation and survival. These molecular abnormalities provide potential therapeutic targets. Oncogene activation or inactivation of tumor suppressor genes leads to disrupted signaling, increased cell growth, decreased inhibitory response, and metastasis. These oncogenes create vulnerability in lung cancer cells and act as therapeutic targets [6]. This understanding has led us to develop targeted therapy focusing on the molecular target in the cancer cell or the tumor microenvironment. Accordingly, targeted therapies are designed to inhibit specific molecular abnormalities ("actionable mutations") that drive tumor growth in certain subsets of NSCLC. However, these mutations are present in only a limited proportion of cases; therefore, chemotherapy remains the mainstay of treatment for a substantial proportion of newly diagnosed patients with NSCLC, accounting for approximately 40% [5]. Receptor tyrosine kinases such as the epidermal growth factor receptor (EGFR), hepatocyte growth factor receptor (c-Met), and anaplastic lymphoma kinase (ALK) are involved in cell growth. They are primary targets for molecular targeted therapies. Currently, investigations are being conducted on inhibitors targeting other targets [7]. Other commonly activated oncogenes in lung cancer include c-ros oncogene 1 (ROS1), human epidermal growth factor receptor 1/Erb-B2 receptor tyrosine kinase 1 (HER1/ERBB1), human epidermal growth factor receptor 2/Erb-B2 receptor tyrosine kinase 2 (HER2/ERBB2), MYC proto-oncogene (MYC), Kirsten rat sarcoma viral oncogene homolog (KRAS), cyclin D1 (CCND1), cyclin-dependent kinase (CDK) 4, and B-cell lymphoma 2 (BCL2).

Targeted therapies have emerged as the cornerstone of treatment for lung cancer over the last two decades, focusing primarily on specific pathways and driver mutations, such as ROS1 fusions, epidermal growth factor receptor (EGFR) and Kirsten rat sarcoma viral oncogene homolog (KRAS) mutations, and anaplastic lymphoma kinase (ALK) rearrangements [8]. The evolution of lung cancer target therapies has transformed the management and prognosis of this once-fatal disease, starting in the early 2000s, when the treatment options were limited, with the development of the first EGFR target therapy. The first significant milestone was the introduction of first-generation tyrosine kinase inhibitors (TKIs), which demonstrated substantial improvements in survival and quality of life compared to chemotherapy, such as gefitinib and erlotinib [9].

Due to resistance, second-generation TKIs, including afatinib and dacomitinib, were developed to overcome these mutations. The objective response rate (ORR) of first- and second-generation TKIS can be up to 60%-70%; nonetheless, resistance is shown after 8-14 months of treatment. This motivated the search for third generations of drugs such as osimertinib and almonertinib [10]. These advances paved the way for specific inhibitors to target other actionable mutations, such as ROS1 fusions and anaplastic lymphoma kinase (ALK) rearrangements, which have significantly increased survival in these patient subgroups [11].

Recently, a breakthrough in targeting mutations previously thought to be unactionable has been made possible by introducing KRAS G12C inhibitors, such as sotorasib. Similarly, the discovery of MET exon 14 skipping mutations has increased the number of targeted treatment choices [12].

This article describes the various therapies targeted against oncogenes causing lung cancer, resistance mechanisms, and possible future advancements.

## Review

Etiologic risk factors for lung cancer

**Figure 1:**
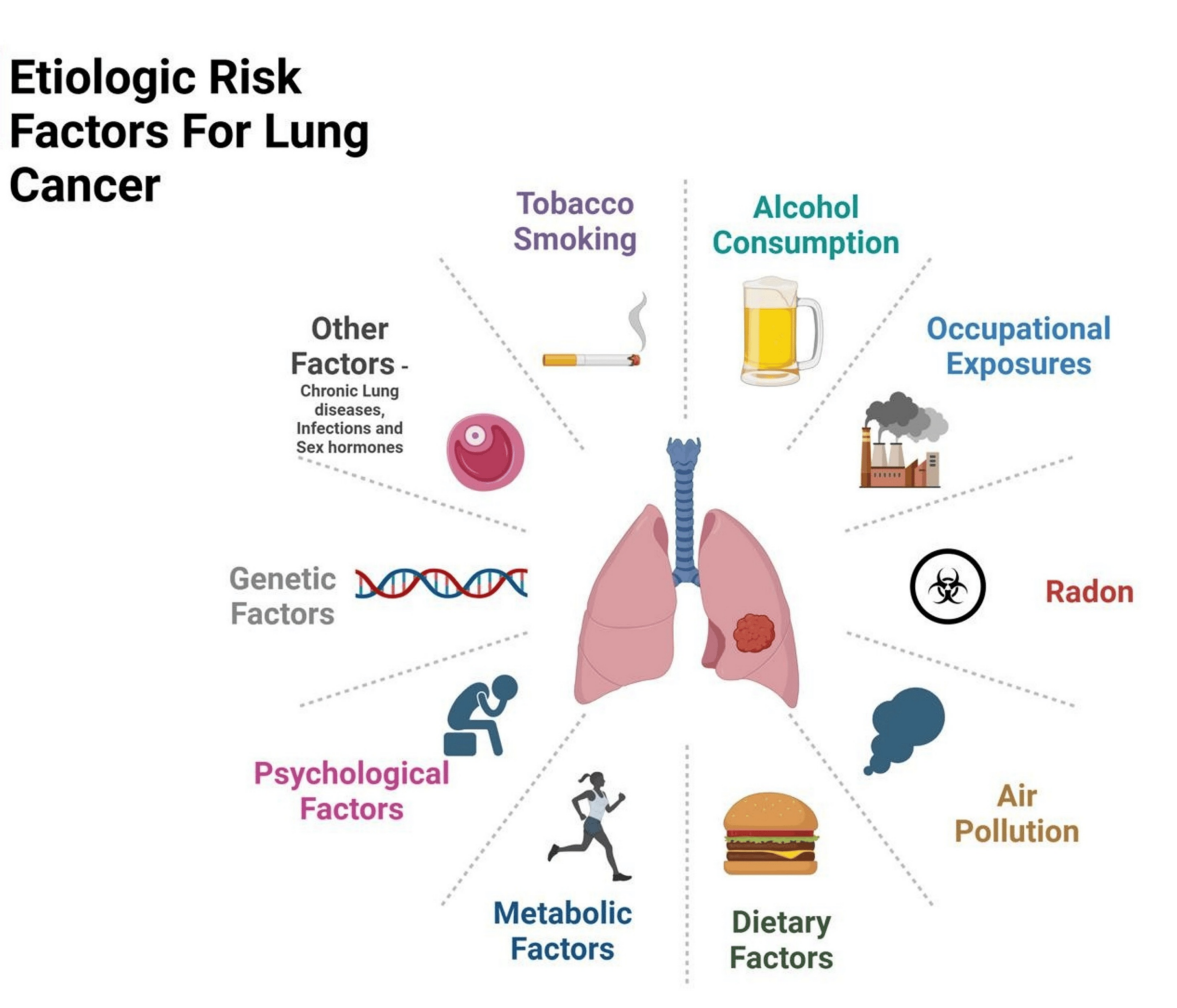
Etiologic risk factors for lung cancer Created with BioRender

Lung cancer risk factors, as demonstrated in Figure 1, include tobacco smoking, genetics, air pollution [13], and exposure to carcinogens such as radon and asbestos [14-16], along with chronic lung diseases and infections, with tobacco smoking being the leading cause [17].

The International Agency for Research on Cancer (IARC) has determined that a cigarette contains over 70 human carcinogens. A positive and linear relationship between exposure to these substances and the development of lung cancer has been established [18,19]. The histological subtypes of lung cancer demonstrate different strengths of association in their relationship with cigarette smoking, with SCC and SCLC showing a stronger relationship, and adenocarcinoma and large cell carcinoma showing a weaker association. Exposure to secondhand smoke has also demonstrated a strong relationship to an increased risk of lung cancer, with the evidence being strongest among partners when compared to childhood exposure [20].

Numerous studies indicate that genetic factors significantly influence lung cancer development, challenging the belief that environmental exposures are the sole cause. A notable finding was that smoking first-degree relatives of patients with lung cancer had a 2.5-fold increased risk compared to controls [21]. Over the past decade, Genome-Wide Association Studies (GWAS) have identified several genetic susceptibility loci linked to lung cancer and its subtypes across different ethnic backgrounds. Specific loci are notably associated with higher cancer risk among Asians [22-24] and Europeans [25-30], and some particularly for adenocarcinoma [31,32], emphasizing the importance of targeting genetic mutations in treatment.

Lung cancer: A prominent global evil

Lung cancer is one of the most common cancers, accounting for 12.4% of all new cancer diagnoses. It is also the prominent cause of cancer-related deaths, making up 18.7% of total fatalities from cancer. While it is the most diagnosed malignancy in men, it is the second most common cancer in women, following breast cancer. In 2022, there were 2.5 million newly diagnosed cases of lung cancer, resulting in 1.8 million fatalities attributed to this disease [33,34].

According to the GLOBOCAN 2022 report, lung cancer ranked as the third most prevalent cancer over one year, comprising 9.1% of all cancer cases. Prevalence over three and five years showed that lung cancer is the fourth most prevalent cancer (only behind breast, colorectal, and prostate cancers), representing 7% and 6% of total cancer cases, respectively.

The prevalence of lung cancer varies across different world regions and is also different between both genders [33,34]. Owing to its vast population, East Asia has the highest incidence, mortality, and prevalence of lung cancer, irrespective of gender, accounting for 50.1% of total global cases, both in absolute numbers; however, interpretation using age-standardized incidence rates (ASIR) suggests that this finding is influenced in part by the region's large population size (ASIR) (Table 1) [33-35]. However, when we compare different regions of the world using ASIR, which allows us to compare different populations with varying distributions of age, Polynesia, a group of Pacific islands, tends to have the second-highest incidence of lung cancer cases in the world regions, despite having a lower number of absolute cases. These could be explained by factors such as increased smoking rates, obesity, poor dietary choices, limited healthcare, and potential exposure to environmental pollutants from past nuclear testing in some areas [36]. However, North America has the highest ASIR for lung cancer incidence among women, which is due to the widespread prevalence of cigarette smoking among North American women [37]. The current estimation is that lung cancer incidence rates in women are fast approaching or even surpassing those in men in several countries in Europe and North America due to increased smoking trends among women in these regions, especially among youngsters [38-40].

**Table 1:** Incidence, mortality, five-year prevalence, and estimated future burden of lung cancer in the world and different UN regions *ASIR: age-standardized incidence rate **ASMR: age-standardized mortality rate ***Prop. (per 100,000): Prevalence proportion is the percentage or proportion of the population affected by lung cancer during a period [33] Source: [33-35]

UN Region	Incidence of Lung Cancer in 2022						Mortality of Lung Cancer in 2022		5-Year Prevalence of Lung Cancer		Estimated Number of New Cases in 2045
	Number of New Cases (Both Genders)	ASIR*(Both Genders)	Number of New Male Cases	ASIR* (Males)	Number of New Female Cases	ASIR* (Females)	Number of Deaths	ASMR**(Both Genders)	Number	Prop ***(Per 100,000)	
World	2,480,675	23.6	1,572,045	32.1	908,630	16.2	1,817,469	16.8	3,221,461	40.9	4,250,779
Eastern Africa	7,549	3.2	4,094	3.9	3,455	2.7	6,974	3.0	11,540	2.5	17,766
Northern Africa	26,280	11.8	21,829	20.6	4,451	3.8	23,694	10.6	35,392	13.9	53,238
Western Africa	4,251	2.1	2,420	2.6	1,831	1.6	3,988	2.0	6,612	1.6	9,425
Central America	10,963	5.4	6,655	7.3	4,308	3.9	10,103	4.9	14,139	7.7	22,263
Northern America	257,284	31.9	128,289	33.8	128,995	30.4	150,675	17.2	325,855	87.3	362,365
South-Eastern Asia	131,184	17.1	91,251	26.0	39,933	9.6	116,366	15.2	178,401	26.2	241,527
Western Asia	61,351	23.3	48,464	38.8	12,887	9.3	55,972	21.5	81,363	28.2	144,089
Northern Europe	73,608	28.0	37,931	30.6	35,677	25.9	52,805	18.7	88,601	82.7	98,535
Western Europe	145,941	31.2	88,993	39.6	56,948	24.0	109,734	22.1	183,833	93.4	176,347
Melanesia	894	11.6	569	15.3	325	8.1	776	10.2	1,313	11.4	1,977
Polynesia	287	37.5	204	54.7	83	21.3	244	31.7	287	41.4	524
Middle Africa	2,055	2.3	1,311	3.2	744	1.6	1,904	2.2	3,082	1.6	4,710
Southern Africa	9,696	16.8	6,201	25.7	3,495	10.4	8,904	15.7	13,516	19.5	18,615
Caribbean	11,768	17.9	6,993	23.1	4,775	13.5	9,809	14.8	14,499	32.9	18,230
South America	82,575	13.8	48,338	17.9	34,237	10.5	70,934	11.7	107,280	24.5	149,196
Eastern Asia	1,243,931	39.4	783,928	51.4	460,003	28.4	851,876	25.1	1,617,792	100.0	1,946,147
South-Central Asia	129,889	6.6	95,290	9.9	34,599	3.5	118,183	6.1	180,646	8.8	251,526
Eastern Europe	158,147	27.6	116,798	49.8	41,349	11.9	126,840	21.6	207,904	71.2	189,105
Southern Europe	106,610	27.7	73,348	40.8	33,262	16.6	86,190	21.0	129,831	85.6	135,366
Australia/New Zealand	16,222	24.6	9,012	28.0	7,210	21.6	11,313	16.2	19,471	62.9	25,954
Micronesia	190	31.6	127	46.1	63	19.2	185	30.5	104	18.6	358

**Figure 2:**
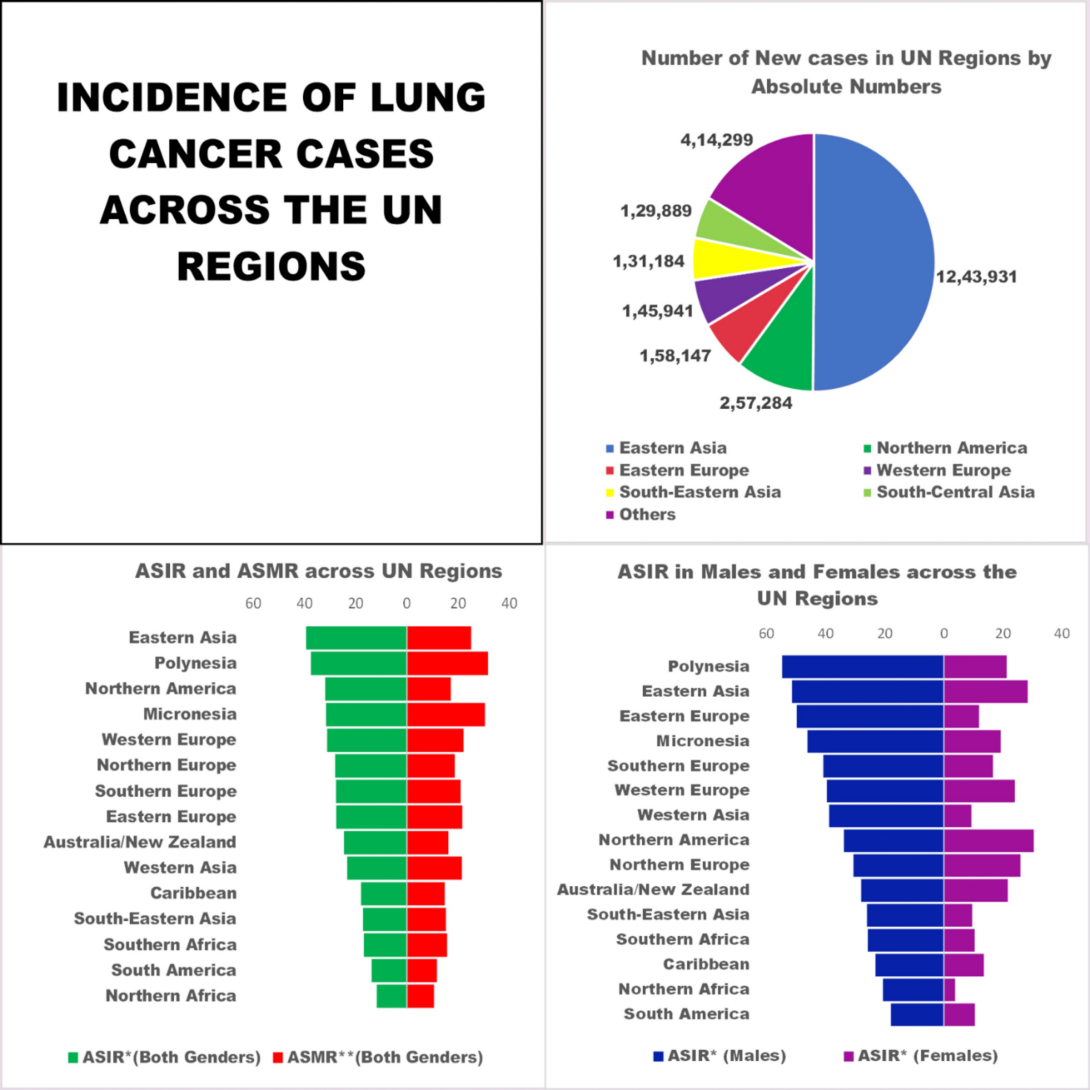
Composite figure of graphs on incidence, ASIR, and ASMR of lung cancer cases across men and women across the United Nations regions ASIR: age-standardized incidence rate, ASMR: age-standardized mortality rate Created with BioRender

Country-wise analysis shows that China has the highest number of lung cancer cases, which is due to China being the top producer and consumer of tobacco [41]. The Chinese government has launched a national strategy called Healthy China 2030 to reduce smoking prevalence to less than 20% by implementing smoke-free laws [42]. Other countries with high lung cancer cases were the United States and Japan, with smoking being the predominant risk factor even in these areas [33,43]. However, compared to Western men, Japanese men had lower lung cancer mortality rates despite their high smoking rates. This mismatch is popularly referred to as the "Japanese smoking paradox." It is partly attributed to genetic factors that may offer resistance to lung cancer in the Japanese male population [44]. Considering the incidence based on ASIR, Hungary has the highest overall incidence rate, and so does the female population. Turkey has the highest ASIR among men, followed by Hungary. Other countries with high ASIR were New Caledonia, Serbia, and French Polynesia, as shown in Figure 2 [33,43]. While smoking was the primary cause of high lung cancer incidence in all these countries, interestingly, the predominant risk factor in New Caledonia was widespread exposure to tremolite asbestos fibers, a type of mineral found in local soil, historically used in whitewashing buildings [45,46].

Another example is that approximately one-third of all patients with lung cancer in East Asia, including China, Japan, and Korea, are non-smokers [47]. In East Asia, never-smokers most often develop adenocarcinoma, and 90% of these people carry oncogenic mutations such as EGFR and ALK [48-51]. Studies have shown different genetic susceptibility of Caucasians and Asians to lung cancer. EGFR mutation is predominant in East Asians (30%) compared to Caucasians (7%), particularly in patients with adenocarcinoma of the lung who are never-smokers [19,52,53]. Similarly, the K-Ras mutation was less prevalent in East Asians (10%) compared to Caucasians (18%), particularly in patients with adenocarcinoma who are smokers [53,54]. Among Polynesians, lung cancer was the most prominent cause of cancer-related mortality among native Hawaiians, and native Hawaiians also suffer worse cancer outcomes, irrespective of their smoking status, due to their genetic makeup [55].

These ethnic variants underscore and reinforce the prominence of genetic susceptibility and oncogene mutations in the burden of lung cancer. While smoking cessation and regular screening play a role in preventing lung cancer, the role of genetic mutations in lung cancer emphasizes the role of personalized therapy targeting oncogene driver mutations in combating lung cancer worldwide. Targeted therapies inhibiting oncogenic mutated kinases are a cornerstone in managing these cases. In this review, we strive to discuss different newly evolving therapies targeted against genetic mutations that cause lung cancer, with more emphasis on therapies targeted against non-small cell lung cancer (NSCLC).

Targeted therapies in lung cancer

**Figure 3:**
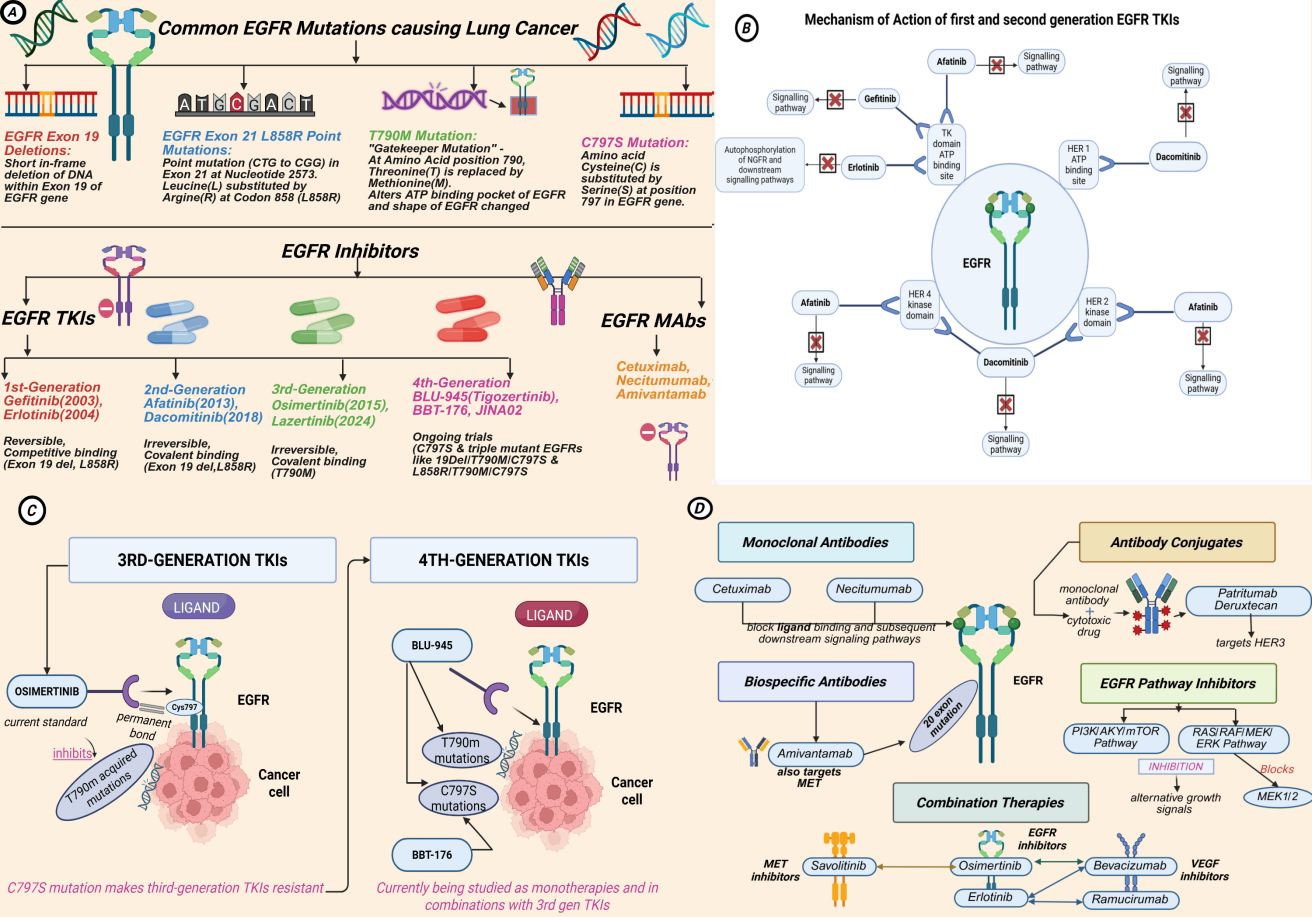
Composite figure with four sections: (A) common EGFR mutations causing lung cancer, (B) MOA of first- and second-generation EGFR TKIs, (C) MOA of third- and fourth-generation EGFR TKIs, and (D) other therapies targeting EGFR EGFR: epidermal growth factor receptor, MOA: mechanism of action, TKI: tyrosine kinase inhibitor Created with BioRender

In Table 2, Table 3, and Figure 3, we discuss all four generations of and other EGFR TKI treatment options for patients with NSCLC and activating EGFR mutations.

**Table 2:** First- and second-generation EGFR TKIs NSCLC: non-small cell lung cancer, EGFR: epidermal growth factor receptor, TKI: tyrosine kinase inhibitor, ATP: adenosine triphosphate, PI3K: phosphoinositide 3-kinase, AKT: protein kinase B, RAS/RAF/MEK: Rat Sarcoma protein, rapidly accelerated fibrosarcoma kinase, mitogen-activated protein kinase kinase signaling pathway proteins, MAPK/ERK: mitogen-activated protein kinase/extracellular signal-regulated kinase, T790M: EGFR resistance mutation, MET/HER2: gene amplifications, PIK3CA: gene mutation in PI3K pathway, EMT: epithelial-mesenchymal transition, SCLC: small cell lung cancer, PFS: progression-free survival, OS: overall survival, PD-1: programmed cell death-1 inhibitor, MOA: mechanism of action

Drug/Target	Indications/Cancer Type	MOA/Pathway	Primary Therapies	Ongoing Clinical Trials	Resistance Mechanisms	Future Advancements	References
Erlotinib	First line for NSCLC with confirmed EGFR mutations (exon 19 deletions or exon 21 L858R), second-line post-platinum therapy, third line in EGFR TKI-naïve patients	Reversible EGFR TKI blocks ATP binding and inhibits autophosphorylation and downstream signaling (PI3K/AKT, RAS/RAF/MEK)	Monotherapy or with bevacizumab	NCT02454933(erlotinib + durvalumab)	T790M mutation (50%), MET/HER2 amplification, PIK3CA mutations, EMT	Combination with MET inhibitors (e.g., savolitinib), liquid biopsy monitoring	[56]
Gefitinib	Advanced metastatic NSCLC with EGFR exon 19 deletions or L858R, post-chemotherapy failure	Selective EGFR TKI inhibits downstream PI3K/AKT, MAPK/ERK signaling	Monotherapy	NCT03832114 (gefitinib + osimertinib)	T790M mutation, HER2/MET amplification, SCLC transformation, EMT	Sequential therapy with Osimertinib, immunotherapy combinations	[57]
Afatinib	EGFR-mutant NSCLC (exon 19, 21), squamous NSCLC post-platinum chemotherapy	Irreversible inhibitor of EGFR, HER2, HER4; blocks downstream signaling	Monotherapy	NCT03755102 (afatinib + osimertinib)	T790M mutation, KRAS/MET alterations, EMT, SCLC transformation	Combination with anti-angiogenics (e.g., bevacizumab), next-generation irreversible TKIs	[57]
Dacomitinib	EGFR-mutated NSCLC (superior PFS/OS versus gefitinib)	Irreversible pan-HER inhibitor (EGFR, HER2, HER4)	Monotherapy	NCT04479306 (dacomitinib + PD-1 inhibitor)	T790M mutation, MET amplification, higher toxicity (rash/diarrhea)	Lower toxicity dosing strategies, combination with MET inhibitors	[58]

**Table 3:** Third- and fourth-generation EGFR inhibitors and other therapies targeting EGFR RTK: receptor tyrosine kinase, ADC: antibody-drug conjugate, MET: mesenchymal-epithelial transition factor, ALK: anaplastic lymphoma kinase, ROS1: c-ros oncogene 1, KRAS: Kirsten rat sarcoma viral oncogene homolog, HER2: human epidermal growth factor receptor 2, FGFR: fibroblast growth factor receptor, VEGFR2: vascular endothelial growth factor receptor 2, BRAF V600E: BRAF gene with valine-to-glutamic acid substitution at codon 600, MEK: mitogen-activated protein kinase kinase, MAPK/ERK: mitogen-activated protein kinase/extracellular signal-regulated kinase pathway, PDGF: platelet-derived growth factor, PD-L1: programmed death-ligand 1, CTLA-4: cytotoxic T-lymphocyte-associated protein 4, MHC: major histocompatibility complex, SHP2: Src-homology 2 domain-containing phosphatase 2, G1202R/G2032R/Y96D: specific resistance mutations at amino acid positions in ALK, ROS1, KRAS, phase I/II/III: stages of clinical trials, FDA: US Food and Drug Administration

Target	Pathway	Primarily Used Targeted Therapies in Lung Cancer	Primary Cancer Type	Ongoing Clinical Trials	Resistance Mechanisms	Future Advancements and Emerging Treatments	References
EGFR	RTK	Third-generation TKIs: osimertinib, lazertinib, nazartinib, almonertinib, mobocertinib (exon 20)	NSCLC (T790M+ or exon 20+)	NCT05442060	C797S mutation (resistance to third-generation TKIs)	Fourth-generation TKIs (BLU-945, BBT-176) in phase I/II trials	[59,60]
EGFR	RTK	Fourth-generation TKIs: BLU-945, BBT-176, poziotinib	NSCLC (C797S+ or exon 20+)	Ongoing	Resistance is not well-defined yet	Targeting C797S mutation and combination therapy	[61-64]
EGFR	Cell surface receptor blockade	Monoclonal antibodies: cetuximab, necitumumab	Squamous NSCLC	Various	EGFR bypass activation	Combination therapy with chemotherapy	[61-64]
EGFR-MET	Dual targeting pathway	Bispecific antibodies: amivantamab (Rybrevant)	NSCLC (exon 20 mutant)	Approved	EGFR and MET pathway activation resistance	Combination with lazertinib to enhance efficacy	[61-64]
EGFR-HER3	ADC	Patritumab deruxtecan (HER3-DXd)	NSCLC (EGFR-resistant cases)	Ongoing	EGFR-independent survival pathways	Used after TKI failure	[61-64]
EGFR downstream pathways	PI3K/AKT, MAPK/ERK pathways	PI3K inhibitors (alpelisib), MEK inhibitors (trametinib, cobimetinib)	NSCLC (TKI-resistant)	Various	Alternative pathway activation	Combination with TKIs for resistance management	[61-64]
EGFR TKI combination therapy	EGFR + VEGF/MET inhibitors	Osimertinib + bevacizumab, erlotinib + ramucirumab, osimertinib + savolitinib	NSCLC	Ongoing trials	Overlapping resistance pathways	Overcoming resistance with dual-targeted approaches	[61-64]

Monoclonal Antibodies (mAbs) and Bispecific Antibodies

Monoclonal antibodies, such as cetuximab and necitumumab, block ligand binding and subsequent downstream signaling pathways by targeting the extracellular domain of EGFR. Their use is supported by the frequent overexpression of EGFR observed in squamous NSCLC, providing a biological rationale for their therapeutic benefit, particularly when combined with chemotherapy or tyrosine kinase inhibitors (TKIs). These combinations may help overcome resistance mechanisms and improve treatment efficacy [65].

Developing bispecific antibodies, such as amivantamab, is another promising treatment. Amivantamab has been approved for advanced NSCLC with EGFR exon 20 insertions based on the CHRYSALIS trial results, which showed an ORR of 40% and a median overall survival (OS) of 22.8 months as a monotherapy. It was also tested in combination with chemotherapy in the same study, where they found no negative impact of this drug. Since early trials showed strong response ratings, the combined therapy is currently being evaluated in the PAPILLON phase III study [66].

Antibody-Drug Conjugates (ADCs)

Antibody-drug conjugates (ADCs) directed toward EGFR present a new therapeutic avenue in NSCLC, especially post-resistance to TKIs, such as osimertinib. These ADCs utilize EGFR-targeting monoclonal antibodies fused to potent cytotoxic payloads to preferentially deliver chemotherapy to neoplastic cells with a dose-sparing effect on normal tissues [67].

Patritumab deruxtecan targets HER3, and studies have shown that it is effective in patients post-osimertinib failure; a phase II trial determined an ORR of 40%, and now it is being investigated for its future potential in combination with osimertinib to overcome resistance [68].

EGFR Pathway Inhibitors

Resistance mechanisms often activate downstream pathways, such as PI3K/AKT/mTOR and RAS/RAF/MEK/ERK, explicitly affecting the effectiveness of osimertinib on EGFR signaling blocking. Inhibitors targeting these downstream pathways have been developed to overcome resistance [69].

PI3K inhibitors targeting PI3K/AKY/mTOR pathways (alpelisib) prevent lung cancer tumor cells from using alternative growth signals when EGFR TKIs fail, which is particularly effective in NSCLC. Alpelisib works better when combined with an EGFR inhibitor since it has limited success as monotherapy [70]. MEK inhibitor targeting the RAS/RAF/MEK/ERK pathway blocks key regulators in the MAPK/ERK signaling cascade: MEK1/2. Trametinib prevents ERK activation and blocks tumor growth, whereas cobimetinib blocks tumor proliferation [71].

Combination Therapies

Combination therapies targeting EGFR with either VEGF or MET inhibitors have become increasingly popular in their ability to overcome resistance to EGFR TKIs in patients with non-small cell lung cancer (NSCLC). These therapies are thought to synergize for tumor suppression by targeting both tumorigenic EGFR-dependent signaling and alternative resistance mechanisms (VEGF and MET) [72].

Osimertinib + bevacizumab is one of the most studied EGFR + VEGF inhibitor combinations. However, its benefits remain inconclusive. A meta-analysis showed that bevacizumab plus osimertinib had a smaller progression-free survival (PFS), OS, and ORR in advanced EGFR mutant NSCLC. However, it resulted in a higher yet manageable occurrence of specific adverse events, including hypertension, nausea, proteinuria, and oral mucositis [73].

On the other hand, erlotinib, a first-generation EGFR TKI, plus bevacizumab as a combined therapy for treatment-naïve patients with NSCLC, has superior PFS compared to erlotinib alone, according to various studies (JO25567, NEJ026, and ARTEMIS). However, there is no evidence of overall survival improvement [74].

RELAY, a phase III trial, studied another VEGF inhibitor-based therapy: erlotinib + ramucirumab. This combination also showed a PFS improvement from 12.4 months with erlotinib alone to 19.4 months after using both drugs [74].

EGFR + MET inhibitor combinations are also gaining attention. The TATTON study evaluated the safety and efficacy of osimertinib plus savolitinib, a MET inhibitor, aiming to overcome MET-driven resistance mechanisms. The results show that patients with MET-amplified, EGFR-mutant NSCLC showed ORRs from 33% to 67% and a PFS between 5.5 and 11.1 months [75].

However, in both of these combined therapies, toxicity management is a critical challenge, as they increase the risk of hypertension, liver toxicity, and bleeding complications.

Driver alterations in all other mutations

Table 4 lists non-EGFR driver alterations commonly targeted in lung cancer management.

**Table 4:** List of non-EGFR driver alterations commonly targeted in lung cancer management RTK: receptor tyrosine kinase, ALK: anaplastic lymphoma kinase, MET: mesenchymal-epithelial transition factor, ROS1: c-ros oncogene 1 receptor tyrosine kinase, KRAS: Kirsten rat sarcoma viral oncogene homolog, HER2: human epidermal growth factor receptor 2, FGFR: fibroblast growth factor receptor, VEGFR2: vascular endothelial growth factor receptor 2, BRAF V600E: BRAF gene mutation with valine-to-glutamic acid substitution at codon 600, EGFR: epidermal growth factor receptor, MEK: mitogen-activated protein kinase kinase, PDGF: platelet-derived growth factor, CTLA-4: cytotoxic T-lymphocyte-associated protein 4, PD-L1: programmed death-ligand 1, TKI: tyrosine kinase inhibitor, ADC: antibody-drug conjugate, SHP2: Src homology 2 domain-containing protein tyrosine phosphatase 2, G1202R/G2032R/Y96D: resistance mutations in ALK, ROS1, and KRAS genes, FDA: US Food and Drug Administration, phase I/II/III: stages of clinical trials evaluating safety and efficacy, CD8+ T cells: cytotoxic T lymphocytes, MHC: major histocompatibility complex

Target	Pathway	Primarily Used Targeted Therapies in Lung Cancer	Primary Cancer Type	Resistance Mechanisms	Future Advancements and Emerging Treatments	References Used in the Table
ALK	RTK	First-generation crizotinib; second-generation alectinib, brigatinib; third-generation lorlatinib	NSCLC	G1202R mutation (causes resistance to first- and second^-^generation ALK inhibitors), presence of bypass pathways (MET and EGFR activation)	A novel ALK-TKI, in the name of TPX-0131, is currently in phase I/II of clinical trials and has been shown to potentially overcome the effects of these mutations	[76-78]
MET	RTK	First-generation crizotinib, second-generation capmatinib, tepotinib	NSCLC	Tyrosine kinase undergoing domain mutation on MET receptor	Third-generation MET inhibitor savolitinib is currently in phase II of clinical trials	[79,80]
ROS1	RTK	First-generation crizotinib, second-generation entrectinib	NSCLC	Bypass signaling via EGFR and KRAS pathways, G2032R mutation in ROS1 receptor tyrosine kinase, causing resistance to first- and second-generation ROS1 inhibitors	Next-generation ROS1 inhibitor repotrectinib is currently in phase II/III of clinical trials	[81-83]
KRAS	RTK	First-generation sotorasib, second-generation adagrasib	NSCLC	Y96D mutation at the drug binding site of KRAS causes resistance to all currently used KRAS inhibitors	TNO155, an SHP2 inhibitor used in combination with KRAS inhibitors, could help prevent any bypassing of signals through a few alternate pathways, and this is still being evaluated in clinical trials; the combination treatment of sotorasib with panitumumab (an EGFR TKI) was approved by the FDA earlier this year in the use of KRAS mutations	[6,84-86]
HER2	RTK	Trastuzumab deruxtecan (antibody-drug conjugate)	NSCLC	HER2 tyrosine kinase domain mutations are known to cause reactivation of downstream pathways, potentially leading to tumor progression	A next-generation, orally administered HER2 TKI named zongertinib is currently being tested for its use in NSCLC (in a phase III clinical trial)	[87-89]
FGFR	RTK	None currently approved for lung cancer treatment	NSCLC	Most commonly caused by gatekeeper mutations in the kinase domain of fibroblast growth factor receptors, FGFR inhibitors are prevented from binding with them to take the necessary effect	Pemigatinib, while already approved to be used in cholangiocarcinoma, is being investigated in a clinical trial (code mentioned on the left) at phase II to see its effectiveness in metastatic phases of NSCLC	[90,91]
VEGFR2	Angiogenesis	Ramucirumab	NSCLC	Activation of other alternative pathways promoting angiogenesis, such as PDGF and EGFR	BMS-690514, a dual-target inhibitor of both HER2 and EGFR pathways, is a treatment option being evaluated for its use in NSCLC- C, found in phase I/IIa of clinical trials	[92,93]
BRAF V600E	RTK	Combination therapy of dabrafenib (BRAF V600E inhibitor) + trametinib (MEK inhibitor), binimetinib	NSCLC	Found to cause resistance via bypass activation of the MEK pathway	PF-07799933, a next-generation treatment option with combined effects of BRAF and MEK inhibitors, is presently being evaluated in phase I of clinical trials; a combination of encorafenib (another BRAF signaling inhibitor) plus binimetinib is currently being clinically trialed in phase II for NSCLC	[94]
CTLA-4 and PD-L1	Immune checkpoint regulators	CTLA 4: ipilimumab, IBI310 PD-1: nivolumab, pembrolizumab, PD-L1: atezolizumab, durvalumab	NSCLC (atezolizumab has also been indicated for use in SCLC)	These tumors can suppress the expression of cell surface receptors (including major histocompatibility complexes), leading to diminished antigen presentation and processing as a part of an immune response	Combination treatment of immune checkpoint inhibitors with nivolumab and ipilimumab (action of both anti-CTLA-4 and anti-PD-L1 antibody function) is currently being studied for its effect in NSCLC (in phase II/III of clinical trials) as a mechanism to bypass the resistance posed by single-agent therapy and promote CD8+ T-cell response toward the cancerous tissues and potentially reducing the risk of immunosuppression; it has already been approved as a treatment option for malignant melanomas	[95,96]

ALK

Anaplastic lymphoma kinase (ALK) is a gene that, through the effect of receptor tyrosine kinase (RTK), typically supports tissue homeostasis and neural development by promoting cell proliferation and growth. However, in the context of lung cancer, ALK most frequently becomes oncogenic through genetic rearrangements with the echinoderm microtubule-associated protein (EML4) forming the EML4-ALK fusion gene, which activates ALK tyrosine kinase and promotes carcinogenesis through uncontrolled cell division and multiplication [76]. This is found to be responsible for approximately 4% of NSCLC cases, mostly in adenocarcinomas, and is characteristically seen in younger patients, as well as non-smokers [97].

**Figure 4:**
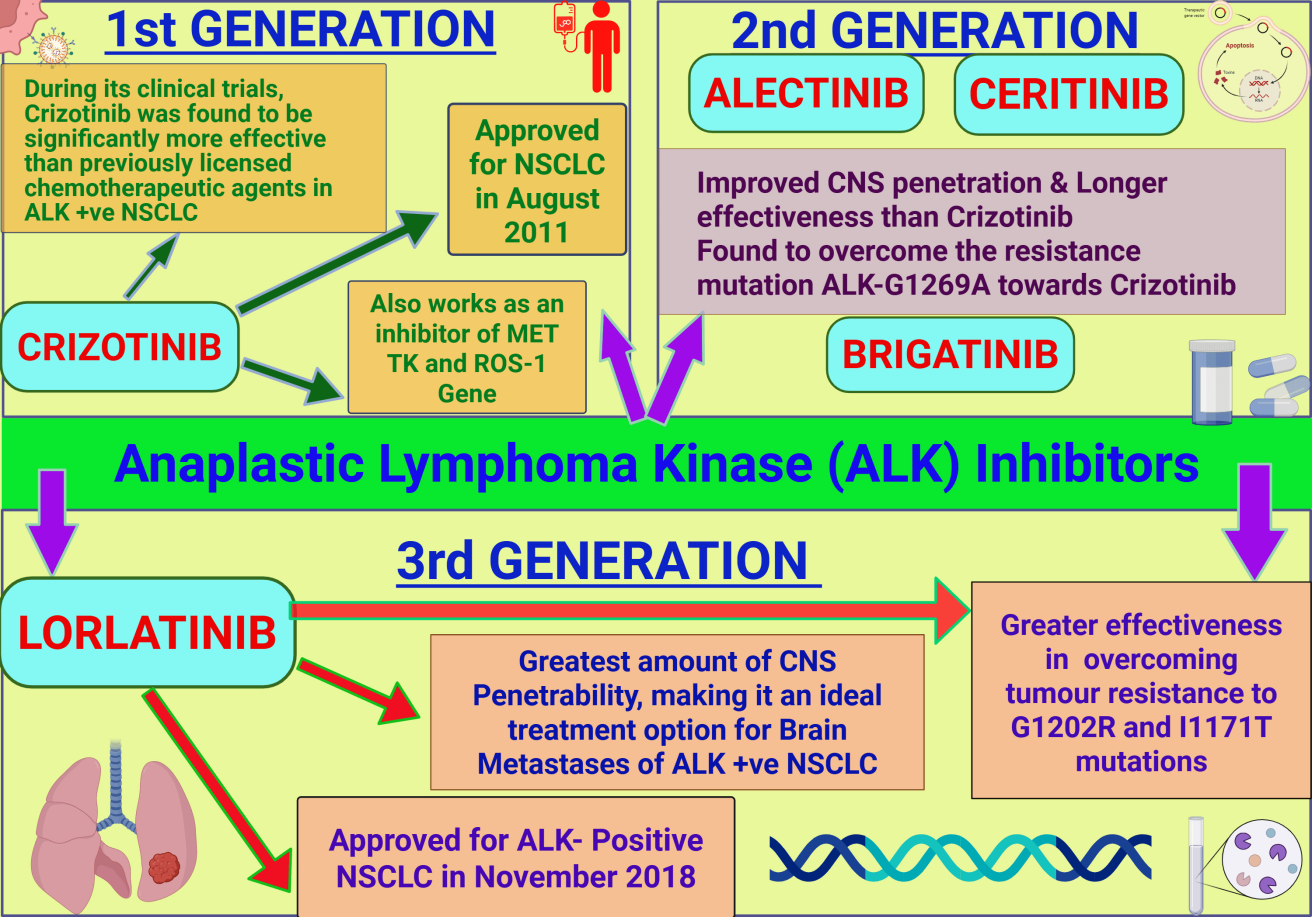
ALK inhibitor therapies ALK: anaplastic lymphoma kinase, NSCLC: non-small cell lung cancer, MET: mesenchymal-epithelial transition factor, TK: tyrosine kinase, ROS1: c-ros oncogene 1, CNS: central nervous system Created with BioRender

RTK inhibitors have been crucial in the treatment of ALK-positive lung cancers. As demonstrated in Figure 4, first-generation therapies, including crizotinib, showed significant efficacy, which was eventually limited by resistance mutations such as G1202R and I1171T [77]. Second-generation inhibitors, such as alectinib and brigatinib, provide improved central nervous system penetration and durability of response. Lorlatinib, a third-generation ALK inhibitor, has also been efficacious in overcoming the earlier-mentioned resistance mechanisms [78].

MET

Like ALK, the mesenchymal-epithelial transition (MET) gene also encodes an RTK, leading to cell growth and survival in a similar mechanism. In lung cancer, oncogenesis most commonly occurs because of a specific mutation in the MET gene called exon 14 skipping [79]. This can prevent the degradation of the MET tyrosine kinase receptor, which can cause uninhibited signaling and drive tumorigenesis. This happens in around 3% of patients with NSCLC, and while MET driver mutations are also associated with adenocarcinomas, they are more common in the elderly population [98]. Approved targeted inhibitors of MET tyrosine kinase include tepotinib and capmatinib, which have demonstrated efficacy in patients with MET exon 14 skip mutations, offering a tailored therapeutic approach for patients with NSCLC (Table 4). However, resistance mechanisms, including secondary mutations, continue to be a barrier to effective targeted treatment, and research is currently being undertaken into combination therapies and next-generation agents, such as savolitinib, to overcome this limitation [80].

FGFR

Fibroblast growth factor receptors (FGFRs) are another class of RTKs known to precipitate about 3% of NSCLC cases when specific alterations in the FGFR gene, such as amplification or formation of fusion genes, occur [90]. Although there are no treatments currently licensed specifically for lung cancers that target FGFR, this is currently being studied extensively, and existing treatments in this class, such as pemigatinib (presently approved therapy for advanced cholangiocarcinoma), are being trialed as therapeutic options in advanced NSCLC with FGFR2 mutations [91].

BRAF V600E

This mutation in the BRAF proto-oncogene plays a cardinal role in controlling and signaling cell growth in typical cases. In the presence of a mutation such as BRAF V600E, where the amino acid valine is substituted by glutamic acid at amino acid location 600 in the BRAF gene, this causes erratic and uninhibited cell division and leads to the onset of various types of cancers, including malignant melanoma, NSCLC, papillary thyroid carcinoma, and colorectal cancer [99]. Around 2% of NSCLCs have a BRAF mutation, most of which are of the V600E variant. This specific mutation is most frequently found in the elderly population and those with a history of heavy smoking [99]. There is currently a combination treatment that uses a BRAF inhibitor along with a MEK inhibitor (to potentially prevent bypassing mechanism of tumor resistance to a sole BRAF inhibitor) approved by the FDA in the management of advanced NSCLC, containing dabrafenib (targets BRAF) and trametinib (MEK) (Table 4) [94].

HER2

Human epidermal growth factor receptor 2 (HER2) is an RTK protein encoded by the ERBB2 gene and is usually responsible for tissue repair and growth. However, specific genetic alterations characteristically drive oncogenesis in malignancies affecting the breast, ovaries, and lungs, usually through their overexpression and amplification. This leads to HER2 receptors binding together and forming dimers, which signals cell proliferation's acceleration and malignant transformation [87]. In the case of lung cancers, HER2 mutations lead to nearly 2% of cases and tend to more commonly cause adenocarcinoma NSCLCs and are found mainly in older female patients who have never smoked in usual instances [100]. Trastuzumab deruxtecan is a targeted therapy with notable efficacy and is FDA-approved for managing advanced NSCLCs driven by HER2-specific mutations [88]. Two orally active pharmaceuticals, poziotinib and zongertinib, are currently being evaluated in clinical trials for their efficacy in targeting a specific HER2 mutation called an exon 20 insertion [89].

KRAS Oncogene

The KRAS oncogene belongs to the RAS family. KRAS gene mutations are seen in about 15%-25% of adenocarcinoma cases. Among patients with locally advanced or metastatic NSCLC, sotorasib was the initial FDA-approved targeted treatment against this mutation. They were also associated with the KRAS G12C mutation. DT2216 (a PROTAC) and sotorasib were given jointly for better results. It was evaluated only in preclinical studies, not in human clinical trials. Specifically, the researchers tested this combination in animal xenograft models to assess its antitumor activity. No patient-based clinical trial data were reported. Also, it showed an outstanding improvement in sotorasib's antitumor effectiveness [6].

Furthermore, adagrasib and afatinib prevented the development of cancers with KRAS mutations; however, it was noted that afatinib encountered the KRAS G12C inhibitor's versatile resistance. This was later discovered to be overcome by SHP2 inhibitors (TNO155), and in combination with sotorasib, trials found that the results had demonstrated improved tumor growth inhibition. However, the presence of add-on mutations and bypass track pathways is a challenge affecting the success of different treatments. Co-mutations with genetic alterations, acquired resistance, alterations in the signaling pathways of KRAS, and secondary KRAS mutations were a few reasons that led to the evolution of resistance to the KRAS (G12C) blocking agents.

Future studies should focus on evolving multifaceted therapy options, including immune checkpoint inhibitors, inhibitors of tyrosine kinase, KRAS upstream inhibitors, and multi-kinase inhibitors against co-mutations, for a good future outcome [85].

ROS1

The ROS1 gene, a tyrosine kinase receptor, plays a vital role in activating various signaling pathways. ROS1 rearrangement is rare and is only present in 0.9%-2.6% of NSCLC cases [81].

Crizotinib is a small-molecule inhibitor of receptor tyrosine kinase (RTK) c-Met, ALK, and ROS1. Crizotinib was first approved for ROS1 in the United States in 2016. Another approved drug is entrectinib. Although crizotinib and entrectinib showed a high success rate, they also showed the evolution of acquired ROS1-resistant mutations during treatment [82]. The efficacy in patients with brain metastases was also limited. It gave intracranial responses, which were short-lasting [101].

Repotrectinib is a newer ROS1 inhibitor, considered less vulnerable to secondary resistance, and has more CNS activity than previous drugs. Studies have shown that repotrectinib has long-lasting clinical activity in the brain and is widely used in clinical practice over crizotinib in patients with brain metastasis [83].

VEGFR2

Vascular endothelial growth factor (VEGF) is mainly associated with angiogenesis, and it has tyrosine kinase domains that help in downstream signaling. The Food and Drug Administration (FDA) approved bevacizumab as the first anti-VEGF monoclonal antibody in 2006 [102]. Ramucirumab is another monoclonal antibody blocking VEGFR2 and is widely used in treating metastatic lung cancer and other solid tumors in combination with different anticancer therapies [92].

Moreover, other advanced studies are being conducted. Various studies have documented increased expression levels of heat shock proteins (HSPs) in cancer cells when exposed to different stimuli, such as chemotherapy, inhibition of tyrosine kinases, oxidative stress, and hyperthermia. BMS-690514 is another vital inhibitor of the VEGF receptor, acting by decreasing the expression of HSP40, in non-small cell lung cancer (NSCLC) cells [93].

PD-L1

**Figure 5:**
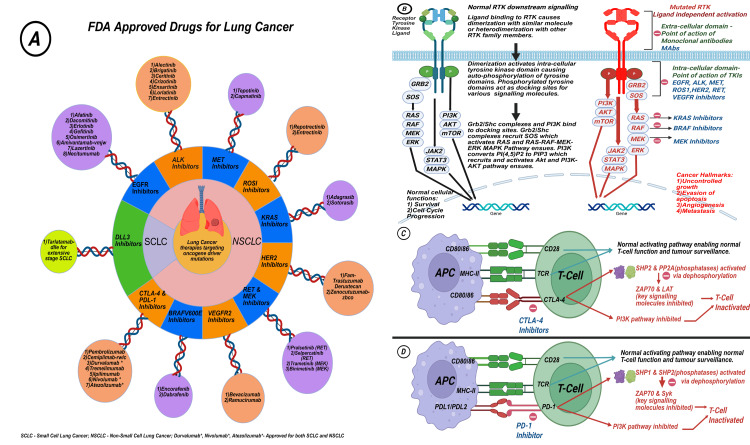
Composite figure with four sections: (A) overview of therapies targeting oncogene driver mutations and FDA-approved drugs, (B): mechanism by which mutated RTKs cause lung cancer and site of action of various RTKIs, (C) mechanism by which CTLA-4 receptor activation promotes cancer, and (D) mechanism by which PD-1 receptor activation promotes cancer FDA: US Food and Drug Administration, RTK: receptor tyrosine kinase, RTKI: receptor tyrosine kinase inhibitor, PD-1: programmed cell death-1 inhibitor, ALK: anaplastic lymphoma kinase, MET: mesenchymal-epithelial transition factor, ROS1: c-ros oncogene 1, KRAS: Kirsten rat sarcoma viral oncogene homolog, HER2: human epidermal growth factor receptor 2, MEK: mitogen-activated protein kinase kinase, VEGFR2: vascular endothelial growth factor receptor 2, CTLA-4: cytotoxic T-lymphocyte-associated protein 4, PD-L1: programmed death-ligand 1, NSCLC: non-small cell lung cancer, SCLC: small cell lung cancer, RET: REarranged during Transfection, APC: adenomatous polyposis coli, TCR: T-cell receptor Created with BioRender

PD-1 is an immunoglobulin superfamily type I transmembrane glycoprotein crucially expressed on activated T cells, as demonstrated in Figure 5. When a tumor cell faces an immune system attack, it has various methods to resist the immune system. At times, they secrete immunosuppressive substances, promoting immune cells with a suppressive nature and expressing them at the membrane surface. Selected inhibitors against specific receptors can block this immunosuppression, creating an anti-tumor effect [95].

When discussing targeted therapies against PD-L1, pembrolizumab is a monoclonal antibody proven to have anti-tumor effects in progressed NSCLC. Pembrolizumab, when given as a single drug as a first-line treatment option, is beneficial for cancers with high expression of PD-L1 (≥50%) [95].

However, the response rate was better with nivolumab plus ipilimumab among people with cancers that expressed PD-L1 in a later study about advanced NSCLC [96].

DLL3

DLL3 acts as an inhibitory ligand and is prevalent in small cell lung cancer (SCLC) treatment. As of now, the treatment methods focusing on DLL3 are different, including antibody-drug conjugates (ADCs), bispecific T-cell engagers (BiTEs), and chimeric antigen receptor (CAR) T-cell therapies. Among the three, the DLL3-targeted BiTEs have held potential clinical interest [103].

Tarlatamab has demonstrated advancement-free survival compared to basic care in a phase II trial; its biologics license application (BLA) is currently under FDA review. Various studies have focused on learning tarlatamab's clinical efficiency [103]. Another promising development is the induction of pluripotent stem cell (iPSC)-derived immune cells [104].

Resistance mechanisms to targeted therapy in lung cancer

In the current scenario, resistance to these therapies is unavoidable. It may be primary (pre-existing genetic alterations prevent the tumor from having the initial desired response to treatment), adaptive (the initial effect of therapy is not sustained due to a cancer cell's plastic counter response), and acquired (new mutations or cellular transformations emerge in response to therapy, causing failure of treatment) [105].

Commonly identified mechanisms include bypass signaling activation, histological transformation, tumor microenvironment and immune evasion, and epigenetic modifications.

Bypass Signaling Activation

Cancer cells can circumvent targeted inhibition by activating alternative signaling pathways. MET or HER2 amplification KRAS mutations are well-documented resistance mechanisms as they activate and/or sustain downstream signaling pathways independent of the targeted oncogene [106].

Histological Transformation

NSCLS cells can undergo phenotypic changes to become more resistant. Epithelial-to-mesenchymal transition enables tumor cells to become more invasive and resistant to apoptosis. NSCLC tumors treated with TKIs may evolve into small cell lung cancer, which is histologically distinct and more aggressive and, hence, does not respond to EGFR inhibitors [107].

Tumor Microenvironment and Immune Evasion

Inter- and intra-tumor heterogeneity is crucial in mediating resistance to targeted therapy [105]. Hypoxia and fibroblast-mediated stromal interactions support drug resistance by promoting cell survival signals [108]. Some tumors can activate immune checkpoints by upregulating PD-L1 expression, leading to immune evasion [109].

Epigenetic Modifications

Epigenetic changes, such as DNA methylation, histone modifications, and non-coding RNA regulation, can alter gene expression and contribute to resistance [108].

**Figure 6:**
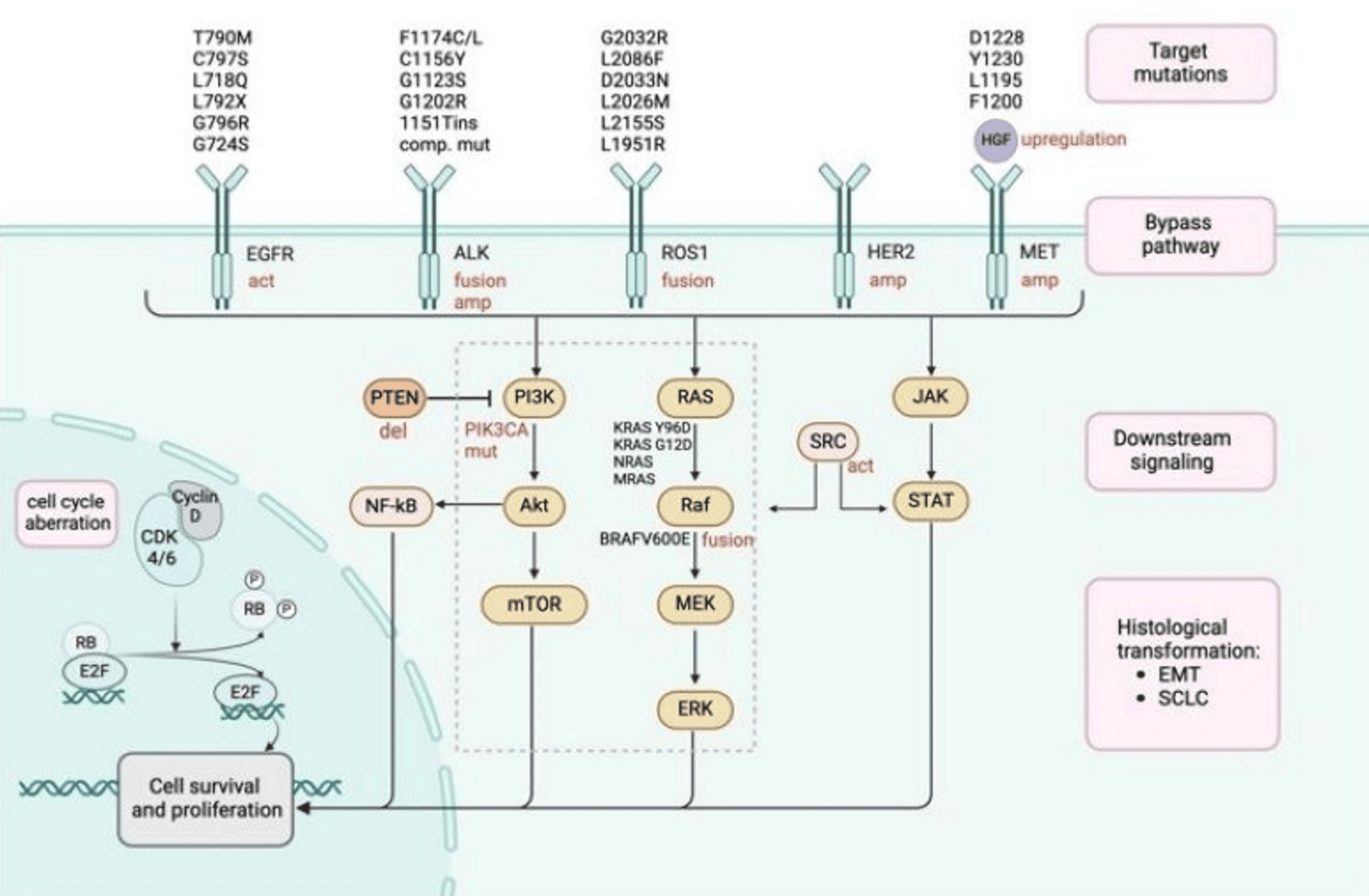
Resistance mechanisms EGFR: epidermal growth factor receptor, ALK: anaplastic lymphoma kinase, ROS1: c-ros oncogene 1, HER2: human epidermal growth factor receptor 2, MET: mesenchymal-epithelial transition factor, NF-κB: nuclear factor-kappa B (NF-κB), MEK: mitogen-activated protein kinase kinase, ERK: extracellular signal-regulated kinase, CDK: cyclin-dependent kinase, EMT: epithelial-to-mesenchymal transition, SCLC: small cell lung cancer, RAS: rat sarcoma, JAK: Janus kinase, SRC: SRC proto-oncogene, non-receptor tyrosine kinase, STAT: signal transducer and activator of transcription, mTOR: mechanistic target of rapamycin, HGF: hepatocyte growth factor Created with BioRender

Here, we discuss the resistance mechanisms of the individual targets encountered with some of the main therapies mentioned above, which are also demonstrated in Figure 6.

EGFR: T790M and C797S

Despite the efficacy of EGFR tyrosine kinase inhibitors, resistance inevitably develops within 12-18 months of treatment [110]. The T790M mutation increases the kinase affinity for ATP, thereby limiting the binding of ATP-competitive kinase inhibitors. Osimertinib, the third generation of this category of drugs, has established itself as the standard first-line treatment for advanced-stage EGFR-mutated NSCLC based on superior efficacy and favorable toxicity profile compared to earlier-generation TKIs [111]. While resistance to first- and second-generation TKIs is mostly target-dependent, resistance to osimertinib is highly heterogeneous. When osimertinib was used as second-line treatment, 10%-26% of cases of resistance were due to tertiary EGFR C797S mutation [112,113]. Moreover, in the FLAURA trial, when osimertinib was administered as a front-line treatment, the C797S mutation accounted for only 7% of the resistance mechanisms, and there was no evidence of EGFR T790M [111].

Bypass Signaling Activation

The most frequent mechanism underlying bypass signaling activation involves overexpression or amplification of the MET proto-oncogene, encountered in 15% and 19% of patients, after completion of osimertinib therapy, when osimertinib was used as first- and second-line treatment, respectively [114,115]. HER2, another tyrosine kinase receptor, activates the downstream PI3K and MAPK pathways, enabling survival signaling independent of EGFR [116]. Additional mechanisms of bypass-mediated resistance have been identified, including fusion events of established drivers of NSCLC, such as FGFR, BRAF, ROS1, REarranged during Transfection (RET), NTRK, and ALK [109,117]. Oncogenic fusions occur at a relatively higher frequency for osimertinib than earlier EGFR TKIs [118].

Downstream Signaling Pathways

The RAS-MAPK and PI3K pathways are the main downstream effector pathways of EGFR that can become reactivated and mediate resistance. The RAS-MAPK pathway can be reactivated by multiple mechanisms influencing each pathway point. NRAS, KRAS, and BRAF V600E mutations and gain of wild-type NRAS or KRAS have been associated with resistance to EGFR TKI in first- and subsequent-line therapy [119,120].

Furthermore, within the PI3K pathway, genetic alterations in PIK3CA (E454K, E542K, R88Q, N345K, and E418K) and phosphatase and tensin homolog (PTEN) deletion may cause bypass activation of the PI3K pathway. Mutations of PIK3CA, which codes for the catalytic subunit p100α protein, frequently coexist with other oncogenic driver mutations, contrary to the mutual exclusivity of most oncogenic driver mutations [121].

Cell cycle aberrations encompassing alterations in genes encoding cyclin D1, cyclin D2, cyclin E1, cyclin-dependent kinase (CDK) 4, and CDK6, and a frameshift deletion in the gene encoding the CDK inhibitor 2A may also play a role in acquiring resistance to osimertinib [115].

Phenotypic Transformation

NSCLC cells can transition into SCLC, characterized by RB1 and TP53 inactivation, which renders EGFR TKIs ineffective [122,123]. Epithelial-to-mesenchymal transition (EMT) has been noted in patients with NSCLC with acquired resistance to gefitinib or osimertinib. EMT enhances migration, invasion, and drug resistance, often driven by TGF-β signaling that activates SMAD2 [124]. Increased NOTCH-1 signaling has been linked to this transformation [125]. In patients with EMT, transcriptional regulators such as Zeb1, Snail, and TWIST1 can be potential targets for future therapies [126,127].

ALK

As with early-generation EGFR TKIs, acquired resistance to the first-generation ALK TKI, crizotinib, typically emerged within a year of starting treatment. ALK-dependent resistance occurs through several somatic kinase domain mutations (187), including point mutations L1196M, C1156Y, L1152R, 1151Tins, G1202R, and S1206Y, and ALK gene copy number gain [128]. Bypass tracks such as EGFR, KRAS, and KIT also confer off-target resistance [129].

For second-generation ALK TKIs, ceritinib, alectinib, and brigatinib, ALK resistance mutations account for over half of patients developing resistance. While the frequency and spectrum of these resistance mutations depend on the specific ALK inhibitor used [130], G1202R is the most frequent mutation that drives resistance to second-generation ALK TKIs. Patients receiving sequential ALK inhibitor therapy may develop compound mutations. Some important ALK resistance mutations seen are F1174C/L, C1156Y, and G1123S with ceritinib; I1171T/N/S, L1196M, and G1269S with alectinib; and G1206C, E1210K+S1206C, and E1210K+D1203N with brigatinib [131]. MEK, SRC, KIT, IGF-1 R, and EGFR activation can also trigger downstream and bypass signaling independent of ALK in cases treated with ceritinib.

The third-generation ALK TKI, lorlatinib, is active against most secondary ALK mutations; however, a diverse array of compound ALK mutations, L1196M/D1203N, F1174L/G1202R, and C1156Y/G1269A, may confer resistance in more than one-third of the cases [132]. In keeping with lorlatinib's efficacy against wild-type ALK and the sequential development of compound mutations, first-line treatment with lorlatinib could prevent on-target resistance [133]. Moreover, BIM 11 with missing polymorphisms, the low minimum allele frequency of the EML4-ALK rearrangement, and MYC amplification are potential causes of primary resistance to ALK TKIs. Primary resistance is observed in 5%-7% of patients after crizotinib, 9% of patients after ceritinib, and 25% of patients after lorlatinib use [134].

ROS1

Resistance mechanisms to ROS1 inhibitors mirror those of ALK inhibitors. Point mutations within the ROS1 kinase domain, like the G2032R solvent front mutation, cause steric interference to TKI binding, rendering crizotinib, entrectinib, and lorlatinib ineffective [135]. Other point mutations have also been identified, with later-generation ROS1 inhibitors showing activity against most of them. Hence, repotrectinib may be preferable as first-line therapy [136]. However, ROS1 L2086F is known to resist repotrectinib and similar agents. Resistance can also arise through bypass signaling involving KRAS, NRAS, and MAP2K1 mutations, MET amplification, NF1 loss, and EGFR activation [137].

HER2

Poor outcomes with pan-HER TKIs, potentially due to HER2 amplification, prompted the development of HER2 selective inhibitors [138]. However, resistance to them may develop through bypass activations of EGFR and MET. While antibody-drug conjugates (ADCs) show promise in HER2-mutant NSCLC, diverse and complex resistance mechanisms exist. These include structural transformation and reduction in the number of HER2 receptors, decreased internalization of HER2, increased lysosomal pH, diminished activity of lysosomal enzymes, and changes in proteins involved in signaling pathways [88].

BRAF

Resistance in BRAF V600E-mutant NSCLC has been shown to arise due to BRAF splice variants (e.g., p61-BRAF V600E) that facilitate dimerization, bypassing the need for upstream activation [139]. Secondary mutations in KRAS (KRASG12D upon dabrafenib treatment) and NRAS (NRASQ61K after dual BRAF/MEK inhibition), upregulation of EGFR, and MEK activation are frequently observed in post-treatment BRAF-mutant NSCLC [99]. Activation of the MAPK pathway by MAP3K8 or COT and the PI3K/AKT pathway via RTK overexpression, PTEN loss, or mutations in PI3KC and AKT promotes resistance. Cell cycle aberrations, such as amplification of CCND1 and cyclin-dependent kinase 4 (CDK4) and loss of function of CDKN2A, may also play a role in resistance to RAF inhibitors [139].

MET

Resistance to MET TKIs, used in MET exon 14, altered NSCLC, and MET-amplified, EGFR-mutated NSCLC may be due to mutations in the kinase domain. D1228 and Y1230, as well as L1195 and F1200, have been commonly reported to provide resistance to type 1 and 2 TKIs [140]. This may be overcome by switching between type 1 and 2 TKIs. HGF upregulation and binding to the intact ligand binding domain increase MET activation and diminish the binding of type 2 TKIs. Thus, a combination with antibodies against HGF or against the ligand may be helpful in such cases [141]. Resistance often correlates with MET amplification, alternative EGFR pathways, and KRAS mutations [142].

Future advancements and emerging therapies

Liquid Biopsies

**Figure 7:**
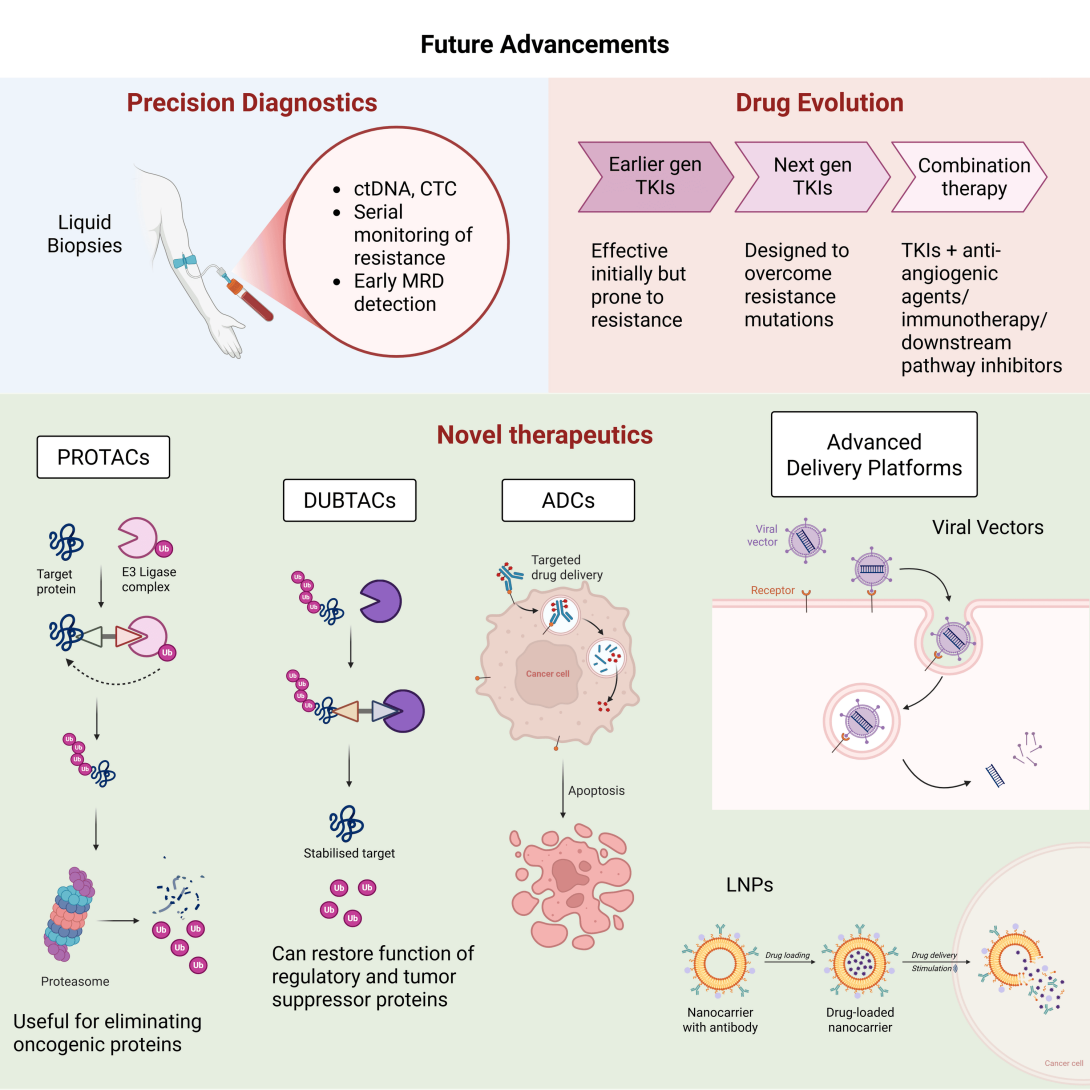
Future advancements in lung cancer therapies ctDNA: circulating tumor DNA, CTC: circulating tumor cell, MRD: minimal residual disease, TKI: tyrosine kinase inhibitor, ADC: antibody-drug conjugate, PROTACs: proteolysis-targeting chimeras, DUBTACs: deubiquitinase-targeting chimeras, LNPs: lipid nanoparticles Created with BioRender

Liquid biopsies analyze circulating tumor DNA (ctDNA), exomes, and circulating tumor cells (CTCs) from a blood sample. Liquid biopsies have several key applications in lung cancer treatment and management, including early detection, treatment selection, monitoring response, and minimal residual disease (MRD) detection (Figure 7).

Liquid biopsies are currently used in standard NSCLC care mainly for molecular profiling and treatment selection, and sometimes for monitoring resistance mutations. However, applications such as early detection and minimal residual disease (MRD) detection using ctDNA remain largely investigational. In particular, ctDNA-based MRD detection is currently used mainly in research and clinical trials rather than routine clinical practice.

In the development of non-invasive methods based on liquid biopsy analysis of ctDNA, in patients with cancer, a fraction of the ctDNA is tumor-derived and, as a result, referred to as ctDNA. As a basic principle, analysis of ctDNA has the major advantage of correlating with alterations specific to the tumor. A study by Phallen et al. (2017) laid the foundation for the commercial use of liquid biopsies by proving that ctDNA can detect early stages of NSCLC and ultra-sensitive sequencing using Targeted Error Correction Sequencing (TEC-Seq), which was used to detect low-frequency mutations. In a cohort of 200 patients with early-stage cancer and 44 healthy controls, 42 of the 200 had a confirmed diagnosis of NSCLC, and ctDNA was measured in these patients. NSCLC-specific data showed 59% detection in early-stage lung cancer, along with 100% specificity in healthy controls. The method was successful even in tumors less than 1 cm. On the other hand, high-depth sequencing remains expensive for routine use [143].

In a diagnostic accuracy study with a cohort of 100 patients with NSCLC, tissue and plasma samples were used to demonstrate that ctDNA can reliably detect cancer-associated mutations in blood samples, matching tissue biopsy results with 90% concordance in the specific case of NSCLC. EGFR exon 19 deletions or L858R mutations were detected with a sensitivity of 70%-90% that varied by stage, with minimal false positives indicating close to 100% specificity. The study distinguished liquid biopsies as a clinically valid tool for initial EGFR testing and real-time resistance monitoring. However, it showed lower sensitivity in early-stage NSCLC, specifically stages I-II [144].

Liquid biopsies can detect rare mutations such as MET exon 14 skipping and RET fusions. Pre-treatment ctDNA levels have been used to identify low-risk patients who may benefit from sequential TKI treatment. ctDNA levels have been shown to identify patients with significantly improved survival rates post-treatment [145,146]. Serial detection provides insight into tumor burden, eliminating the need for repeating tissue biopsies. Several studies have found a significant association between quantitative changes in ctDNA, cancer response to the targeted therapy, and the prognosis of NSCLC.

Minimal residual disease (MRD) is represented by early tumor development or relapse. Liquid biopsies can be used for early cancer treatments through their advantage of detecting micrometastatic disease and early abnormalities. A prospective longitudinal cohort study was conducted, where serial ctDNA detected relapse months earlier than imaging and predicted higher relapse risk [147].

Liquid biopsies, specifically ctDNA, have become an indispensable tool in the management of EGFR-mutated NSCLC, particularly for patients treated with first- and second-generation EGFR TKIs. In summary, they have enabled non-invasive detection of EGFR mutations, real-time monitoring, and early identification of resistance mechanisms.

Epigenetic Drugs

Resistance to EGFR TKIs remains a significant challenge in NSCLC; epigenetic therapies are emerging onto the scene as a promising strategy of resistance reversal and a method of drug sensitivity restoration. Epigenetic therapies target gene expression modifications brought by DNA methylation and histone acetylation. These processes contribute to cancer progression and, in the case of NSCLC, the development of resistance to EGFR TKIs.

There are multiple classes of epigenetic drugs (Figure 7); the most prominent are DNA methyltransferase inhibitors (DNMTis), histone deacetylase inhibitors (HSACis), and EZH2 inhibitors, which target histone methylation. Going into more detail, DNMTIs reverse the hypermethylation of tumor suppressor genes, such as CDKN2A and adenomatous polyposis coli (APC), thus restoring TKI sensitivity. DNMTi monotherapy showed minimal clinical response in trials involving NSCLC.

Using DNMTi as a key player in combination therapies has yielded promising results. A phase I/II study of combined epigenetic therapy utilizes azacitidine and entinostat, which are DNA methylation and histone deacetylation inhibitors. The study reflected that nearly half of the patient cohort achieved disease control. Tumor biopsies taken post-treatment concluded that tumor suppressor genes CDKN2A and APC were re-expressed, which was in harmony with the clinical benefit [148].

AI and Machine Learning, Predictive Modeling, and the Use of Radiogenomics in Lung Cancer Screening and Aiding in Personalized Therapy

The use of artificial intelligence and machine learning has been slowly emerging as a key player in revolutionizing the prediction of resistance and personalized therapy. It has been shown to be very beneficial in predicting resistance mechanisms and identifying early relapse. As a landmark paper, the study by Chabon et al. (2020) demonstrated a method in which circulating tumor DNA (ctDNA) profiling combined with machine learning can predict EGFR TKI resistance in NSCLC. The machine learning model predicted T790M emergence with 92% accuracy months before progression. It also uncovered NF1 loss and TB1 mutations as the main driving forces of resistance in T790M-negative tumors. The machine learning model could also detect MET amplification in ctDNA, which was confirmed later using tissue biopsies [149].

Another study leveraged the ctDNA mutation burden, fragment size, and copy number alterations at baseline to train a machine learning model to avoid the need for longitudinal sampling. Baseline ctDNA features predicted progression risk with higher accuracy. The main predictive values utilized by the machine learning model were short ctDNA fragments, TP53 co-mutations, and high EGFR VAF. This study reflected how machine learning predictions can assist in guiding decisions regarding treatment [150].

Limitations and challenges

Limitations of Targeted Therapies in Lung Cancer

The advent of targeted therapies has dramatically changed the prognosis for patients with actionable mutations in non-small cell lung cancer (NSCLC) by improving survival rates. These therapies, however valuable, do have some significant obstacles, including drug resistance, toxicity, interpatient variability, and economic access [151]. Such obstacles limit the effectiveness of targeted treatments on the patient's health over an extended period, suggesting a necessity to investigate further the gaps in overcoming resistance, optimizing toxicity measures, and providing wider access to these therapies.

Resistance Mechanisms

Therapeutic resistance develops after an initial response to a targeted therapy, almost invariably resulting in disease progression. Resistance can be primary (intrinsic) when the tumor never responds to treatment from the start despite harboring a targetable mutation [152]. This is commonly attributed to the genetic heterogeneity of the tumors, where different subpopulations of cancer cells within the same individual have different genetic profiles and treatment sensitivity [153]. A case in point is our understanding of EGFR mutations and their response to therapy, such as reservoir host responses, which exhibit weak responses toward EGFR tyrosine kinase inhibitors (TKIs) due to exon 20 insertions, making them ineffective as first-line treatment options [8]. Acquired resistance is observed in patients who initially respond to targeted therapy, developing over time through multiple means.

Adverse Side Effects

Compared to traditional chemotherapy, targeted therapies are more selective; however, they can still produce adverse side effects that can decrease adherence to treatment and the patient's quality of life. An example of these adverse effects, known as on-target effects, occurs when the blockade of a specific molecular pathway negatively impacts tissues other than those intended. An acneiform skin rash is commonly caused by EGFR inhibitors, such as erlotinib and osimertinib, due to the blockage of EGFR signaling, which is essential for epidermal homeostasis [153]. Visual complications and peripheral neuropathy are also seen in patients treated with ALK inhibitors crizotinib and lorlatinib due to unintended effects on the peripheral nervous system [152]. Osimertinib, a third-generation EGFR TKI, has also been reported to cause cardiotoxic effects, including QT prolongation and reduced left ventricular ejection fraction, which is suggested to happen by mis-targeted blockade of HER2 in cardiac muscle [8].

Unlike the previously mentioned type of toxicity, immune-oncology treatments bear a unique type of toxicity known as immune-related adverse events (irAEs), which stems from too much activation of the immune system against the body. These toxicities are not restricted to a specific organ and include organ-specific allergic pneumonitis, which can affect around 5% of patients receiving PD-1/PD-L1 inhibitors and can be fatal if left untreated [154].

Financial and Accessibility Barriers

One of the most significant barriers to the widespread implementation of targeted therapies is their high cost, particularly in low- and middle-income countries [154]. Many targeted agents are priced at over $100,000 annually, making them inaccessible to a substantial portion of the global population. Even in high-income countries, insurance restrictions and high copayments can lead to financial toxicity, forcing patients to delay or forgo treatment due to cost concerns [154]. Access to comprehensive biomarker testing also remains a significant limitation, particularly in resource-limited settings. Fewer than 50% of patients with lung cancer worldwide receive comprehensive genomic testing, mainly due to infrastructure and cost barriers [151]. This results in many patients being unable to receive effective targeted treatments, exacerbating global disparities in lung cancer care [154].

## Conclusions

Targeted therapy has been an increasingly studied topic that has revolutionized the management of advanced NSCLC over the past two decades. Regarding their impact on clinical outcomes, their advent has widely proven to be an adequate replacement for traditional chemotherapy, providing a more personalized and safer alternative. This has been found to prolong progression-free survival and improve the quality of life in patients with cancer. This review has highlighted significant gene alterations or driver mutations engendered in NSCLC cases, such as EGFR, ALK, HER2, KRAS, and VEGF.

This review also discussed some of the licensed major pharmacological therapies that specifically target each mutation and, therefore, are shown to be effective in advanced NSCLC, which expresses their respective mutations. Despite their promise, however, there have been a few challenges that have been encountered with these therapies, such as developing acquired drug resistance by cancer cells, managing drug-related toxicity, and addressing financial implications and barriers to their obtainability. The effects of resistance are presently being studied in clinical trials to develop next-generation medication classes that overcome these resistance mechanisms and improve the treatment efficacy of NSCLC tumors.
